# Variability in monthly serum bicarbonate measures in hemodialysis patients: a cohort study

**DOI:** 10.1186/s12882-015-0206-2

**Published:** 2015-12-21

**Authors:** Ravi Patel, William Paredes, Charles B. Hall, Mark A. Nader, Deepak Sapkota, Vaughn W. Folkert, Matthew K. Abramowitz

**Affiliations:** Department of Medicine, Thomas Jefferson University School of Medicine, Philadelphia, PA USA; Division of Nephrology, Department of Medicine, Albert Einstein College of Medicine, Bronx, NY USA; Department of Epidemiology & Population Health, Albert Einstein College of Medicine, 1300 Morris Park Avenue, Ullmann 615, Bronx, NY 10461 USA; The Saul R. Korey Department of Neurology, Albert Einstein College of Medicine, Bronx, NY USA; Division of Nephrology, Department of Medicine, Medstar Georgetown University, Washington, DC USA; Department of Medicine, Interfaith Medical Center, Brooklyn, NY USA

**Keywords:** Bicarbonate, Acidosis, Alkalosis, Variability, Hemodialysis, End-stage renal disease

## Abstract

**Background:**

Some nephrologists have advocated an individualized approach to the prescription of bicarbonate hemodialysis. However, the utility of monthly serum bicarbonate levels for guiding and evaluating such treatment decisions has not been evaluated. We sought to define the variability of these measurements and to determine factors that are associated with month-to-month variability in pre-dialysis serum bicarbonate.

**Methods:**

We examined the monthly variability in serum bicarbonate measurements among 181 hemodialysis patients admitted to a free-standing dialysis unit in the Bronx, NY from 1/1/2008-6/30/2012. All patients were treated with a uniform bicarbonate dialysis prescription (bicarbonate 35 mEq/L, acetate 8 mEq/L). Pre-dialysis serum bicarbonate values were obtained from monthly laboratory reports. Month-to-month variability was defined using a rolling measurement for each time point.

**Results:**

Only 34 % of high serum bicarbonate values (>26 mEq/L) remained high in the subsequent month, whereas 60 % converted to normal (22–26 mEq/L). Of all low values (<22 mEq/L), 41 % were normal the following month, while 58 % remained low. Using the mean 3-month bicarbonate, only 29 % of high values remained high in the next 3-month period. In multivariable-adjusted longitudinal models, both low and high serum bicarbonate values were associated with greater variability than were normal values (β = 0.12 (95 % CI 0.09–0.15) and 0.24 (0.18 to 0.29) respectively). Variability decreased with time, and was significantly associated with age, phosphate binder use, serum creatinine, potassium, and normalized protein catabolic rate.

**Conclusions:**

Monthly pre-dialysis serum bicarbonate levels are highly variable. Even if a clinician takes no action, approximately 50 % of bicarbonate values outside a normal range of 22–26 mEq/L will return to normal in the subsequent month. The decision to change the bicarbonate dialysis prescription should not be based on a single bicarbonate value, and even a 3-month mean may be insufficient.

**Electronic supplementary material:**

The online version of this article (doi:10.1186/s12882-015-0206-2) contains supplementary material, which is available to authorized users.

## Background

Metabolic acidosis is common in patients with end-stage renal disease (ESRD) receiving hemodialysis (HD) [1–3]. The accumulation of acid in the interdialytic period is typically neutralized by HD three times per week with a solution containing a relatively high concentration of bicarbonate buffer. The therapeutic goal for HD patients per KDOQI guidelines is to maintain pre-dialysis serum bicarbonate ≥22 mEq/L [4]. Some would advocate maintaining pre-dialysis bicarbonate levels higher than 22 mEq/L but below a yet-to-be-defined ceiling because studies have shown that both metabolic acidosis and metabolic alkalosis are associated with increased mortality in this population [1, 2].

One approach to achieving a target level of serum bicarbonate is to individualize therapy based on monthly serum bicarbonate levels [5, 6]. However, well-defined protocols are not in widespread use, and the task is made more difficult by the variety of physiological factors affecting acid-base balance in ESRD patients [7, 8]. In addition to the effects of nutrition, volume status, and dialysis adequacy, the delivery of an alkali load may stimulate organic acid generation in some patients, thereby mitigating alkalinization [8, 9]. Furthermore, there may be a great deal of variability in a patient’s bicarbonate level from month to month, but this has not yet been systematically examined. The purpose of this study is to examine the nature of that variability and whether patient characteristics and other biomarkers can predict variability.

We hypothesized that there is substantial intra-individual variability in monthly serum bicarbonate levels. To investigate this, we examined monthly laboratory data for consecutive patients at one of our affiliated dialysis units over a 4-year period. All patients received a uniform dialysis bicarbonate prescription, and no patients prescribed oral alkali were included. We sought to define the magnitude of bicarbonate variability and to determine if this variability was explained by factors such as dialysis treatment characteristics, markers of nutritional status and inflammation, and interdialytic weight gain.

## Materials

### Study population

We conducted this retrospective cohort study in an urban, academically affiliated dialysis center in the Bronx, NY. All patients at this center were treated with a uniform bicarbonate dialysis prescription of 35 mEq/L and a dialysate containing 8 mEq/L acetate during the study period. Our study cohort consisted of all patients receiving HD who were admitted to this facility between January 1, 2008 and June 30, 2012 for at least a 3 month period and did not meet exclusion criteria. Out of 291 patients admitted during this time period, 74 were excluded because they did not receive care at this center for at least three consecutive months, 23 because they were peritoneal dialysis patients, and 11 due to missing data. After the exclusion of two patients who were taking oral sodium bicarbonate, 181 patients were included in the final cohort, with 4104 monthly observations.

### Data collection

Demographic information and medical history were obtained from the patients’ medical records. Information on medical history and phosphate binder type and dose was recorded from the monthly progress note, and epoetin alpha administration was recorded from the dialysis provider’s computerized database. For modeling in regression analyses, phosphate binder use was categorized based on the number of pills prescribed of an acid- (sevelamer hydrochloride) or base- (all other binders) precursor. Information on other prescription medications was recorded at baseline. Cardiovascular disease was defined as a history of coronary artery disease, stroke, peripheral vascular disease, or congestive heart failure. Laboratory data including serum bicarbonate levels were obtained from the patients’ monthly pre-dialysis laboratory reports. In total, 92.6 % of laboratory data were collected on Monday or Tuesday, per standard practice in this dialysis unit. As serum bicarbonate values collected on Monday or Tuesday did not differ from other days of the week (22.6 ± 3.0 mEq/L Monday/Tuesday vs. 22.4 ± 3.6 mEq/L other days, *p* = 0.2), we did not include the day of sample collection in our analyses. Ultrafiltration volume was used as a surrogate for interdialytic weight gain (IDWG), which was defined as the difference between the pre- and post-dialysis weight after a single monthly HD session. We excluded IDWG values <0 and >7 kg as we considered these implausible. Complete data for all covariates of interest was available for 3833 observations in 180 patients. The study protocol was approved by the Institutional Review Board of the Albert Einstein College of Medicine, which granted a waiver of informed consent due to the retrospective, observational design of the study.

### Outcome measures

We first examined scatterplots of monthly bicarbonate values with the bicarbonate value in the subsequent month, and we calculated the intraclass correlation coefficient (ICC) for serum bicarbonate overall and within 6 month time periods. For comparison with another laboratory measure that is highly predictive of clinical outcomes, we repeated these analyses for serum albumin. We classified each serum bicarbonate value into clinical categories using cutpoints that were based on outcomes data in published epidemiologic studies [1, 2, 10], clinical practice guidelines [4], and proposals for clinical practice [3, 11]: low (<22 mEq/L), normal (22–26 mEq/L), and high (>26 mEq/L). We then examined the likelihood of a serum bicarbonate value remaining in the same clinical category on a subsequent month. We repeated this analysis after categorizing patients based on tertiles of mean serum bicarbonate in the first 90 days after admission to the dialysis unit, and within tertiles of the variability index (Variability Value) defined below. We also calculated the mean serum bicarbonate during 3-month intervals, categorized these using the same cutpoints, and calculated the percentage that remained in the same category in the subsequent 3-month period.

We quantified the month-to-month variability in serum bicarbonate by defining a rolling measurement for each time point *i* as follows:

$$ \mathrm{Variability}\ \mathrm{V}\mathrm{alue}\ \left(\mathrm{V}\mathrm{V}\right)=\sqrt{\frac{{\left( BICAR{B}_i\ \hbox{--}\ BICAR{B}_{i-1}\right)}^2 + {\left( BICAR{B}_i\ \hbox{--}\ BICAR{B}_{i+1}\right)}^2}{2}} $$

For each patient, we calculated the mean VV of all observations. The lowest, middle, and highest tertiles of mean VV were used to classify patients as having low, medium, and high variability, respectively.

### Statistical analysis

Baseline laboratory values were defined as the mean of all measurements available during the first 90 days after admission to the dialysis unit. We first characterized the cohort based on baseline serum bicarbonate values to determine if the associations in our cohort were consistent with previous reports. Characteristics of the population categorized by quartiles of serum bicarbonate were compared using *χ*2 for categorical variables and analysis-of-variance or the Kruskal-Wallis test for continuous variables.

Mixed-effects models including time as a random effect were used to examine associations over time of demographic, clinical, and laboratory characteristics with serum bicarbonate and VV, separately. As VV did not fit a Normal distribution, it was log-transformed after adding 1 to the value because VV contained ‘zero’ values: logVV = ln(VV + 1 mEq/L). Demographics, comorbidities, dialysis access type, IDWG, phosphate binder and epoetin alpha prescriptions, and laboratory values related to nutritional status, inflammation, and dialysis adequacy were included in the models based on a priori knowledge of the physiology of acid-base regulation in hemodialysis and previously demonstrated associations with serum bicarbonate. Medications recorded only at baseline, dialysis treatment time, blood flow rate, and dialysate flow rate were considered for inclusion based on a *p*-value <0.20 or >15 % change in other parameters. The association of each covariate with the outcome was tested for linearity using higher-order terms and categorical variables; non-linear associations were modeled by including both linear and quadratic terms in the model, or with categorical variables. As serum creatinine was unavailable in one patient, the multivariable models included 180 patients. The models were fitted with an independent variance-covariance structure of the random effects; using an unstructured covariance matrix did not change the results. To examine whether the association of serum bicarbonate level and monthly variability varied with time, we a priori determined to examine the interaction of serum bicarbonate (in clinical categories) with time in models with logVV as the dependent variable. *P* values for interaction with time were not calculated for other covariates as these were considered exploratory analyses. Statistical analyses were performed using Stata software, version 13.1 (Stata Corporation, College Station, TX, USA). A *p*-value <0.05 was considered statistically significant.

## Results

### Patient characteristics

The baseline characteristics of the study participants are shown in Table 1. The mean age was 56 years, 41 % were female, and the majority were Black or Hispanic (44 and 40 %, respectively). Comorbidities were common, and diabetes mellitus was the most common cause of ESRD. The initial access type was a catheter in 41 % of patients, although nearly all (62 of 75) eventually transitioned to either an arteriovenous fistula (AVF) or an arteriovenous graft (AVG). Phosphate binders were prescribed in 86 % of patients within 120 days of admission to the dialysis unit. Only eight patients were never prescribed a phosphate binder for the duration of follow-up. The mean serum bicarbonate during the first 90 days was 23.0 ± 2.2 mEq/L. Smoking status was not recorded for many patients (39 %). The median follow-up time was 19 months (interquartile range, 10–33) with a range of 4–56 months.

**Table 1:** Baseline characteristics of 181 hemodialysis patients

Age (years)	56.0 ± 16.6
Female – n (%)	74 (40.9)
Race/ethnicity – n (%)	
Black	79 (43.7)
Hispanic	71 (39.2)
Caucasian	22 (12.1)
Other	9 (5.0)
Hypertension – n (%)	176 (97.2)
Diabetes Mellitus – n (%)	108 (59.7)
Cardiovascular Disease – n (%)	116 (64.1)
Chronic Obstructive Pulmonary Disease– n (%)	15 (8.3)
Smoking – n (%)	
Current	24 (13.3)
Former	33 (18.2)
Never	53 (29.3)
Unknown	71 (39.2)
Body Mass Index - kg/m^2^ – n (%)	
Underweight (≤18.49)	4 (2.2)
Normal (18.5 – 24.9)	62 (34.3)
Overweight (25 – 29.9)	64 (35.4)
Obese (≥30)	51 (28.2)
ESRD Etiology – n (%)	
Diabetes	87 (48.1)
Hypertension	45 (24.9)
Glomerulonephritis	12 (6.6)
Polycystic Kidney Disease	3 (1.7)
Lupus	8 (4.4)
HIV	7 (3.9)
Other/Unknown	19 (10.5)
Initial Access Type – n (%)	
Catheter	75 (41.4)
AVF	66 (36.5)
AVG	40 (22.1)
Medication use – n (%)	
Diuretic	25 (13.8)
ACE inhibitor or ARB	63 (34.8)
Beta blocker	133 (73.5)
Calcium Channel Blocker	109 (60.2)
Statin	93 (51.4)
Proton Pump Inhibitor	51 (28.2)
H2 Blocker	10 (5.5)
Phosphate binder– n (%)^a^	
Sevelamer Hydrochloride	76 (42.0)
Calcium Acetate	38 (21.0)
Sevelamer Carbonate	21 (11.6)
Lanthanum Carbonate	6 (3.3)
Calcium Carbonate	6 (3.3)
None	34 (18.8)
Epoetin alpha dose (Units)	
≤5000	57 (31.5)
>5000 – ≤10,000	96 (53.0)
≥10,000	28 (15.5)
Interdialytic weight gain (kg)	2.77 ± 0.93
Dialysis treatment time (min)	228.4 ± 18.0
Blood flow rate (mL/min)	382 ± 31
Dialysate flow rate (mL/min)	603 ± 33
Serum bicarbonate (mEq/L)^b^	23.0 ± 2.2
Serum potassium (mEq/L)^b^	4.6 ± 0.6
Serum albumin (mg/dL)^b^	3.7 ± 0.5
Serum calcium (mg/dL)^b^	8.9 ± 0.6
Serum phosphate (mg/dL)^b^	5.1 ± 1.2
Serum hemoglobin (g/dL)^b^	10.8 ± 1.2
Serum creatinine (mg/dL)^b^	8.0 ± 2.9
WBC (10^3^/mm^3^)^b^	7.5 ± 2.1
nPCR (g/kg/day)^b^	0.82 ± 0.18
spKt/V^b^	1.6 ± 0.28

*Abbreviations*: *BMI* body-mass index; *AVF* arteriovenous fistula; *AVG* arteriovenous graft; *ESRD* end-stage renal disease; *nPCR* normalized protein catabolic rate; *spKt/V* single-pool Kt/V; *WBC* white blood cells

^a^Phosphate binder data reflect prescriptions within the first 120 days after admission to the dialysis unit

^b^Laboratory data are the mean value of all measurements within the first 90 days after admission to the dialysis unit

### Associations of patient characteristics with serum bicarbonate levels

In univariate analyses, baseline serum bicarbonate was not associated with demographics or comorbidities but differed significantly across quartiles by use of an ACE inhibitor or ARB. Patients with higher baseline serum bicarbonate had less IDWG and lower levels of serum potassium, albumin, phosphate, and normalized protein catabolic rate (nPCR) (Additional file 1: Table S1).

In longitudinal unadjusted analyses, older age, heart disease, HD via a tunneled catheter compared to an AVF, single pool Kt/V (spKt/V) ≥1.71 compared to ≤1.51, and higher serum calcium were each associated with higher bicarbonate levels (Table 2). The highest category of sevelamer hydrochloride prescription, greater IDWG, and higher nPCR, albumin, hemoglobin, serum potassium, phosphorus, creatinine, and higher white blood cell (WBC) counts were associated with lower serum bicarbonate over time. After multivariable adjustment, the associations with sevelamer hydrochloride and all laboratory covariates persisted, and there was a significant increase in bicarbonate levels over time.

**Table 2:** Longitudinal associations of patient characteristics with serum bicarbonate levels

	Unadjusted		Multivariable-adjusted	
	Coefficient (95 % CI)	*p*	Coefficient (95 % CI)	*p*
Age (per 10 years)	**0.45 (0.29 to 0.61)**	<0.001	0.07 (−0.10 to 0.25)	0.41
Male	−0.54 (−1.12 to 0.05)	0.07	0.09 (−0.45 to 0.63)	0.75
Race/ethnicity				
White	−0.18 (−1.12 to 0.75)	0.70	−0.61 (−1.45 to 0.23)	0.16
Hispanic/Other	−0.44 (−1.06 to 0.17)	0.16	−0.28 (−0.83 to 0.26)	0.31
BMI				
Overweight	0.06 (−0.63 to 0.74)	0.87	−0.14 (−0.74 to 0.46)	0.66
Obese	−0.01 (−0.74 to 0.72)	0.97	0.16 (−0.48 to 0.80)	0.62
Dialysis access				
AVG	0.22 (−0.20 to 0.64)	0.31	−0.18 (−0.60 to 0.24)	0.40
Tunneled catheter	**0.33 (0.01 to 0.64)**	0.04	0.11 (−0.21 to 0.43)	0.50
Etiology of ESRD				
Diabetes	0.50 (−0.20 to 1.21)	0.16	0.38 (−0.26 to 1.02)	0.24
Other/Unknown	−0.21 (−1.00 to 0.58)	0.60	0.31 (−0.46 to 1.08)	0.43
Cardiovascular Disease	**0.90 (0.31 to 1.48)**	0.003	0.41 (−0.18 to 1.01)	0.17
Phosphate binder				
>6 pills/day acid precursor	**−1.00 (−1.45 to−0.55)**	<0.001	**−0.49 (−0.95 to−0.03)**	0.04
>3−6 pills/day acid precursor	−0.39 (−0.81 to 0.03)	0.07	0.02 (−0.41 to 0.44)	0.9
≤3 pills/day acid precursor	0.002 (−0.38 to 0.39)	0.9	0.27 (−0.13 to 0.66)	0.19
≤3 pills/day base precursor	0.004 (−0.37 to 0.37)	0.9	0.29 (−0.08 to 0.66)	0.12
>3–6 pills/day base precursor	0.04 (−0.35 to 0.43)	0.84	0.36 (−0.04 to 0.75)	0.08
>6 pills/day base precursor	−0.23 (−0.61 to 0.15)	0.24	0.08 (−0.33 to 0.49)	0.70
IDW gain (kg)				
>1–3	**−0.28 (−0.60 to 0.05)**	0.10	0.26 (−0.04 to 0.56)	0.08
>3–7	**−0.65 (−1.01 to−0.30)**	<0.001	0.15 (−0.18 to 0.47)	0.38
Epoetin alpha dose (Units)				
≤5000	0.11 (−0.11 to 0.32)	0.33	−0.01 (−0.20 to 0.19)	0.9
>5000 – ≤10,000	0.16 (−0.09 to 0.40)	0.16	0.05 (−0.18 to 0.28)	0.69
≥10,000	0.04 (−0.30 to 0.38)	0.83	−0.11 (−0.43 to 0.20)	0.47
spKt/V				
1.52–1.70	0.12 (−0.8 to 0.32)	0.25	**0.25 (0.06 to 0.44)**	0.009
≥1.71	**0.31 (0.07 to 0.55)**	0.01	**0.39 (0.16 to 0.62)**	0.001
nPCR (g/kg/day)	**−3.14 (−3.54 to−2.75)**	<0.001	**−1.08 (−1.52 to −0.64)**	<0.001
Serum albumin (g/dL)	**−1.06 (−1.32 to−0.80)**	<0.001	**−0.84 (−1.15 to −0.52)**	<0.001
Hemoglobin (g/dL)	**−0.25 (−0.30 to −0.19)**	<0.001	**−0.14 (−0.19 to −0.08)**	<0.001
K^+^ (mEq/L)	**−1.13 (−1.25 to −1.00)**	<0.001	**−0.70 (−0.83 to −0.57)**	<0.001
Calcium (mg/dL)	**0.74 (0.62 to 0.87)**	<0.001	**0.95 (0.82 to 1.08)**	<0.001
Phosphorus (mg/dL)	**−0.63 (−0.68 to −0.57)**	<0.001	**−0.39 (−0.45 to −0.32)**	<0.001
WBC (10^3^/mm^3^)				
6.26–8.25	−0.14 (−0.36 to 0.09)	0.23	0.26 (−0.04 to 0.56)	0.08
≥8.26	**−0.35 (−0.64 to −0.08)**	0.01	**−0.35 (−0.61 to −0.10)**	0.007
Serum creatinine (mg/dL)	**−0.33 (−0.37 to −0.29)**	<0.001	**−0.14 (−0.19 to −0.08)**	<0.001
Time (months)	0.003 (−0.004 to 0.01)	0.40	**0.02 (0.01 to 0.03)**	<0.001

*Abbreviations*: *CI* confidence interval; *BMI* body-mass index; *AVG* arteriovenous graft; *ESRD* end-stage renal disease; *ACE-I* angiotensin-converting enzyme inhibitor; *ARB* angiotensin receptor blocker; *nPCR* normalized protein catabolic rate; *spKt/V* single-pool Kt/V; *WBC* white blood cells; *IDW* interdialytic weight gain

Models included all variables listed in the Table. Reference categories are female for sex; Black for race/ethnicity; normal/underweight for BMI; arteriovenous fistula (AVF) for dialysis access; hypertension for etiology of ESRD; no binder use for phosphate binder; no epoetin alpha use for epoetin alpha dose; 0-1 kg for interdialytic weight gain; ≤1.51 for spKt/V; ≤6.25 10^3^/mm^3^ for WBC. Bold values indicate p<0.05.

### Monthly serum bicarbonate variability

**Fig. 1:**
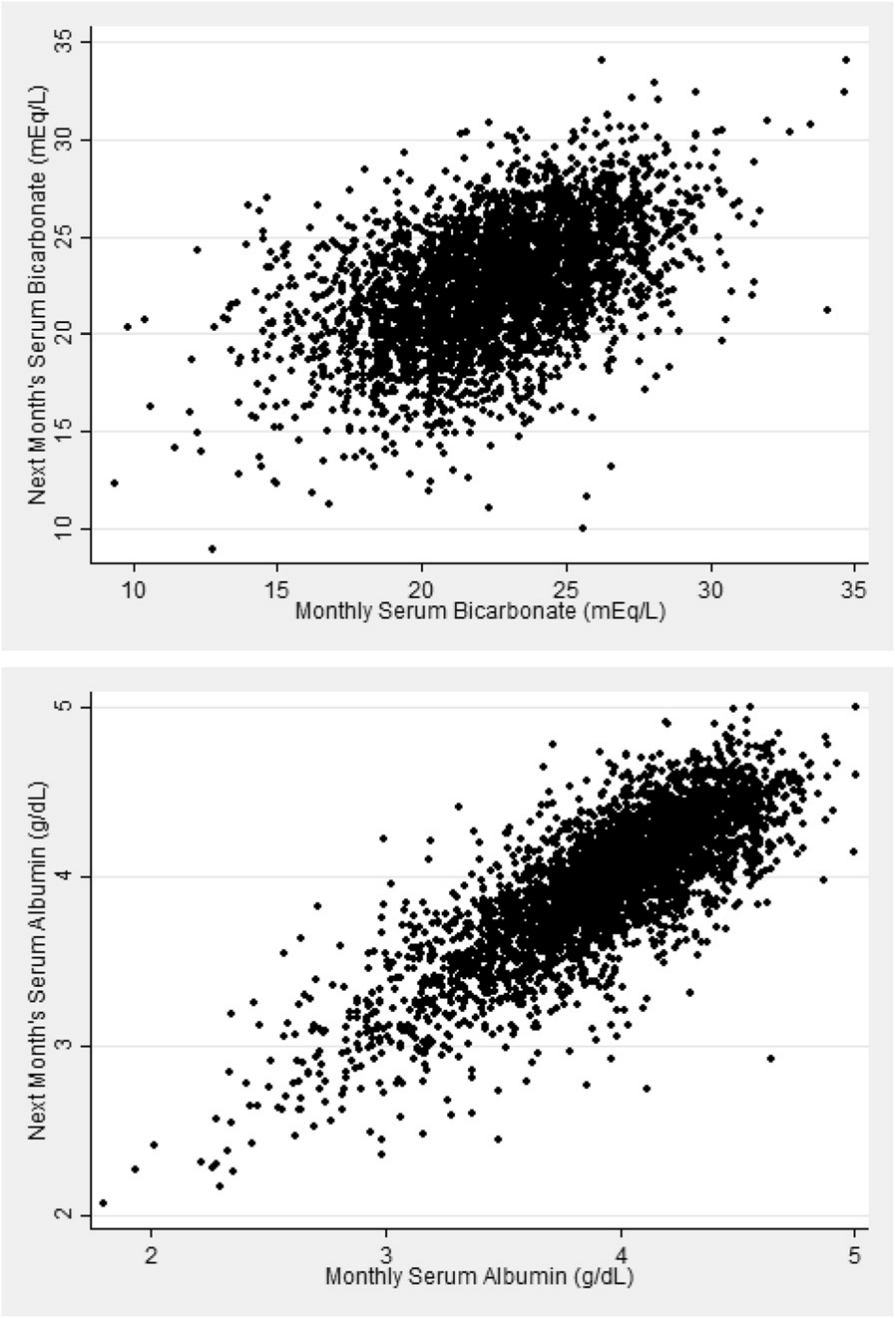
Scatterplots demonstrating the correlation of consecutive monthly measurements. Scatterplots showing the correlation of a monthly measurement (*x-axis*) with the subsequent monthly measurement (*y-axis*) for serum bicarbonate (*top panel*) and serum albumin (*bottom panel*)

**Fig. 2:**
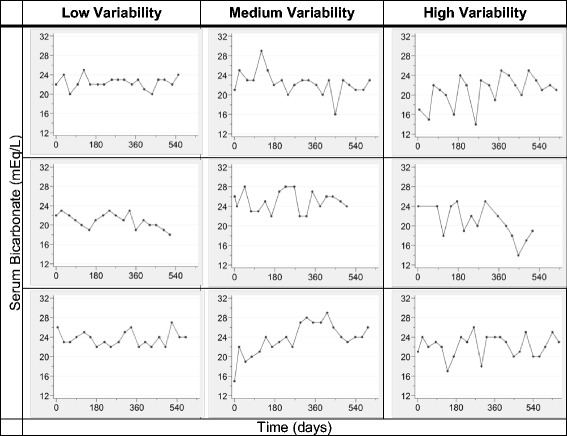
Sample plots demonstrating levels of variability. Representative graphs of serum bicarbonate over time are shown for 3 patients in each of the 3 variability categories. Low, medium, and high variability were defined based on tertiles of the mean value of VV (Variability Value) for each patient during follow-up: Low (mean VV ≤2.18 mEq/L), Medium (mean VV = 2.19 to 2.75 mEq/L), and High (mean VV ≥2.76 mEq/L)

**Fig. 3:**
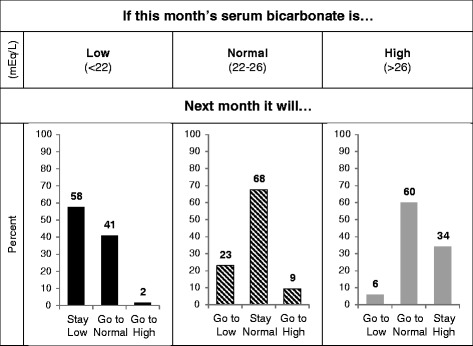
Predicting next month’s bicarbonate level. For a single monthly serum bicarbonate measurement falling within the low (<22 mEq/L), normal (22–26 mEq/L), and high (>26 mEq/L) categories defined by clinical cutpoints, the percentage of measurements in the following month that remain in the same category or change category are shown. Percentages may not sum to exactly 100 % due to rounding

**Fig. 4:**
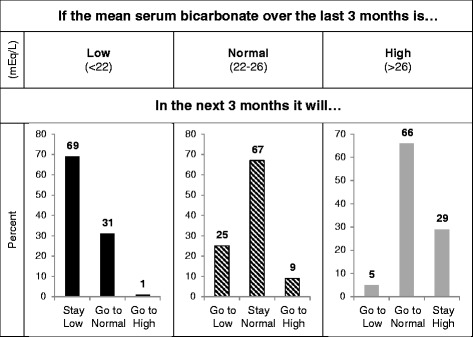
Predicting the next 3-month mean serum bicarbonate level. For each mean serum bicarbonate calculated over a 3-month period and falling within the low (<22 mEq/L), normal (22–26 mEq/L), and high (>26 mEq/L) categories defined by clinical cutpoints, the percentage of mean values in the following 3-month period that remain in the same category or change category are shown. Percentages may not sum to exactly 100 % due to rounding

The ICC for serum albumin was substantially greater overall and in each 6-month interval than for serum bicarbonate (Table 3). Scatterplots also demonstrated greater correlation of a monthly measurement with the subsequent month’s value for serum albumin than for serum bicarbonate (Fig. 1). We next examined the variability of serum bicarbonate measurements in more detail. Representative plots of serum bicarbonate levels over time for patients in each of the 3 variability categories are shown in Fig. 2. Substantial month-to-month variability is apparent, and appears greatest in the highest tertile of VV. Overall, 58 % of low serum bicarbonate values (<22 mEq/L) remained low in the subsequent month (Fig. 3, Additional file 1: Table S2). Of all high values (>26 mEq/L), 60 % were normal the following month, while only 34 % remained high. Of all normal values (22–26 mEq/L), 68 % remained normal the following month. These results were largely unchanged even when examined within tertiles of the 90-day mean serum bicarbonate (Additional file 1: Figure S1). Only among patients in the lowest tertile of 90-day mean serum bicarbonate were low values more likely to remain low than to convert to normal the following month. Regardless of 90-day bicarbonate tertile, high values were more likely to convert to normal than to remain high. Using the mean bicarbonate value over 3-month time periods, low and normal values were equally likely to remain in the same category (69 and 67 %, respectively), whereas only 29 % of high values remained high (Fig. 4). When examined within VV tertiles, bicarbonate values outside the normal range appeared most likely to change category among patients with high variability (i.e. the highest VV tertile) (Additional file 1: Figure S2).

**Table 3:** Intraclass correlation coefficients for serum bicarbonate and albumin

	Number of patients	Number of observations (bicarbonate/albumin)	Serum bicarbonate	Serum albumin
Overall	181	4104/4099	0.38	0.68
0–6 months	181	1160/1156	0.34	0.68
>6–12 months	156	849/849	0.47	0.74
>12–18 months	117	609/609	0.51	0.78
>18–24 months	93	526/525	0.53	0.80
>24–30 months	70	373/373	0.57	0.73
>30–36 months	53	277/277	0.41	0.64
>36 months	36	310/310	0.54	0.61

### Associations of patient characteristics with monthly bicarbonate variability

In multivariable-adjusted longitudinal models, both low and high serum bicarbonate values were associated with greater monthly variability (Table 4). Older age and higher hemoglobin were associated with lower variability, and variability decreased with time. Use of an AVG (compared with AVF), use of epoetin alpha, and higher serum creatinine were associated with greater variability. Non-linear associations were noted with nPCR, serum K^+^, and calcium, and there was a trend toward lower variability with use of any phosphate binder compared with none.

**Table 4:** Multivariable-adjusted associations of clinical and laboratory characteristics with serum bicarbonate variability over time

	Overall		≤12 months		>12 months	
	Coefficient (95 % CI)	*p*	Coefficient (95 % CI)	*p*	Coefficient (95 % CI)	*p*
Age (per 10 years)	**−0.03 (−0.04 to −0.01)**	0.009	**−0.03 (−0.05 to 0.00)**	0.03	0.00 (−0.03 to 0.03)	0.9
Male	−0.04 (−0.09 to 0.02)	0.19	**−0.09 (−0.16 to −0.01)**	0.02	0.02 (−0.05 to 0.09)	0.65
Race/ethnicity						
White	−0.03 (−0.11 to 0.06)	0.53	−0.03 (−0.13 to 0.08)	0.64	−0.07 (−0.17 to 0.04)	0.22
Hispanic/Other	−0.03 (−0.09 to 0.03)	0.31	−0.04 (−0.11 to 0.03)	0.24	0.00 (−0.07 to 0.08)	0.9
BMI						
Overweight	0.01 (−0.05 to 0.07)	0.78	0.02 (−0.06 to 0.09)	0.65	0.02 (−0.05 to 0.10)	0.56
Obese	−0.02 (−0.09 to 0.04)	0.52	−0.01 (−0.09 to 0.07)	0.83	−0.01 (−0.10 to 0.07)	0.77
Dialysis access						
AVG	**0.06 (0.00 to 0.11)**	0.04	0.01 (−0.06 to 0.09)	0.75	**0.07 (0.00 to 0.14)**	0.04
Tunneled catheter	0.04 (−0.01 to 0.09)	0.15	0.00 (−0.07 to 0.07)	0.9	**0.19 (0.09 to 0.28)**	<0.001
Etiology of ESRD						
Diabetes	−0.03 (−0.09 to 0.04)	0.41	−0.04 (−0.12 to 0.04)	0.33	0.03 (−0.06 to 0.11)	0.54
Other/Unknown	−0.06 (−0.14 to 0.01)	0.11	−0.08 (−0.18 to 0.01)	0.09	0.00 (−0.09 to 0.10)	0.9
Cardiovascular Disease	0.05 (−0.01 to 0.11)	0.13	0.06 (−0.02 to 0.13)	0.13	0.04 (−0.04 to 0.11)	0.32
Phosphate binder						
>6 pills/day acid precursor	−0.03 (−0.11 to 0.05)	0.50	0.05 (−0.07 to 0.17)	0.39	−0.09 (−0.21 to 0.04)	0.19
>3–6 pills/day acid precursor	−0.05 (−0.12 to 0.03)	0.21	0.04 (−0.07 to 0.14)	0.50	−0.12 (−0.25 to 0.01)	0.06
≤3 pills/day acid precursor	**−0.09 (−0.16 to −0.02)**	0.01	−0.06 (−0.15 to 0.02)	0.16	−0.12 (−0.24 to 0.01)	0.07
≤3 pills/day base precursor	−0.04 (−0.11 to 0.02)	0.21	−0.02 (−0.10 to 0.06)	0.60	−0.06 (−0.18 to 0.06)	0.31
>3–6 pills/day base precursor	−0.07 (−0.14 to 0.00)	0.07	0.00 (−0.10 to 0.09)	0.95	**−0.14 (−0.26 to −0.01)**	0.03
>6 pills/day base precursor	**−0.08 (−0.15 to 0.00)**	0.04	−0.06 (−0.16 to 0.05)	0.30	−0.11 (−0.23 to 0.01)	0.07
Epoetin alpha dose (Units)						
≤5000	**0.05 (0.02 to 0.09)**	0.005	−0.02 (−0.07 to 0.04)	0.57	**0.10 (0.05 to 0.15)**	<0.001
>5000 – ≤10,000	0.03 (−0.02 to 0.07)	0.23	0.01 (−0.05 to 0.07)	0.81	0.03 (−0.04 to 0.09)	0.44
≥10,000	**0.06 (0.00 to 0.12)**	0.04	−0.02 (−0.10 to 0.06)	0.65	**0.16 (0.07 to 0.25)**	<0.001
Serum bicarbonate (mEq/L)						
<22	**0.12 (0.09 to 0.15)**	<0.001	**0.17 (0.13 to 0.22)**	<0.001	**0.08 (0.03 to 0.12)**	0.001
>26	**0.24 (0.18 to 0.29)**	<0.001	**0.23 (0.16 to 0.31)**	<0.001	**0.25 (0.18 to 0.32)**	<0.001
Serum albumin (g/dL)	0.03 (−0.02 to 0.09)	0.25	**0.07 (0.00 to 0.15)**	0.04	−0.04 (−0.13 to 0.04)	0.32
nPCR (g/kg/day)	**−0.42 (−0.73 to −0.10)**	0.009	−0.23 (−0.80 to 0.33)	0.42	**−0.46 (−0.84 to −0.07)**	0.02
nPCR^2^ ((g/kg/day)^2^)	**0.19 (0.04 to 0.34)**	0.01	0.09 (−0.20 to 0.37)	0.55	**0.21 (0.04 to 0.38)**	0.02
spKt/V	0.04 (−0.02 to 0.10)	0.18	0.04 (−0.04 to 0.11)	0.35	0.04 (−0.04 to 0.12)	0.34
K^+^ (mEq/L)	−0.18 (−0.36 to 0.00)	0.05	−0.10 (−0.36 to 0.16)	0.47	−0.23 (−0.48 to 0.02)	0.07
K^+2^ ((mEq/L)^2^)	**0.02 (0.00 to 0.04)**	0.05	0.01 (−0.02 to 0.03)	0.49	0.02 (0.00 to 0.05)	0.05
Hemoglobin (g/dL)	**−0.02 (−0.03 to −0.01)**	0.002	**−0.04 (−0.05 to −0.02)**	<0.001	0.00 (−0.02 to 0.02)	0.9
Calcium (mg/dL)	**−0.34 (−0.57 to −0.12)**	0.003	**−0.48 (−0.74 to −0.22)**	<0.001	−0.09 (−0.55 to 0.38)	0.71
Calcium^2^ ((mg/dL)^2^)	**0.02 (0.01 to 0.03)**	0.002	**0.03 (0.01 to 0.04)**	<0.001	0.01 (−0.02 to 0.03)	0.65
Phosphorus (mg/dL)	0.01 (0.00 to 0.02)	0.09	0.00 (−0.02 to 0.01)	0.64	**0.03 (0.01 to 0.04)**	<0.001
Serum creatinine (mg/dL)	**0.01 (0.00 to 0.02)**	0.03	0.01 (0.00 to 0.02)	0.19	0.01 (0.00 to 0.02)	0.11
WBC (10^3^/mm^3^)	0.01 (0.00 to 0.01)	0.17	0.01 (0.00 to 0.02)	0.13	0.00 (−0.01 to 0.01)	0.79
IDW gain (kg)						
>1–3	0.01 (−0.05 to 0.07)	0.8	−0.03 (−0.12 to 0.05)	0.41	0.04 (−0.05 to 0.12)	0.38
>3–7	0.02 (−0.04 to 0.09)	0.48	−0.03 (−0.12 to 0.06)	0.55	0.07 (−0.02 to 0.15)	0.15
Time (months)						
>3–6	**−0.05 (−0.11 to 0.00)**	0.04	−0.05 (−0.11 to 0.00)	0.06	….	
>6–12	**−0.09 (−0.14 to −0.04)**	<0.001	**−0.10 (−0.15 to −0.04)**	0.001	….	
>12–24	**−0.12 (−0.18 to −0.07)**	<0.001	….		ref	
>24	**−0.14 (−0.20 to −0.07)**	<0.001	….		0.00 (−0.04 to 0.04)	0.88

*Abbreviations*: *CI* confidence interval; *BMI* body-mass index; *AVG* arteriovenous graft; *ESRD* end-stage renal disease; *nPCR* normalized protein catabolic rate; *spKt/V* single-pool Kt/V; *WBC* white blood cells; *IDW* interdialytic weight gain

Models included all variables listed in the Table. Reference categories are female for sex; Black for race/ethnicity; normal/underweight for BMI; arteriovenous fistula (AVF) for dialysis access; hypertension for etiology of ESRD; no binder use for phosphate binder; no epoetin alpha use for epoetin alpha dose; 22–26 mEq/L for serum bicarbonate; 0–1 kg for interdialytic weight gain; ≤3 months for time in the Overall and ≤12 months models, and >12–24 months in the >12 months model. Bold values indicate p<0.05.

Time-stratified analyses suggested that a number of associations were modified by time and were not dependent on the choice of time cutpoint (6 or 12 months) (Table 4, Additional file 1: Table S3). The variability of low bicarbonate values appeared to decrease with time, whereas that of high values did not (*p* = 0.02 for interaction with time). Male sex was associated with lower variability only in the early period after dialysis initiation. A dialysis access other than an AVF and epoetin alpha use were associated with greater variability, and phosphate binder use with lower variability, only after 6–12 months of follow-up. Associations of a number of other laboratory parameters with variability were modified by time, but not in a consistent direction.

## Discussion

Acid-base homeostasis in dialysis patients is a complicated process affected by multiple variables, all potentially leading to marked variation in an individual patient’s pre-dialysis serum bicarbonate. Indeed, we found significant variability in monthly serum bicarbonate measurements among patients receiving HD with a uniform bicarbonate dialysis prescription, such that a single measurement considered high or low based on clinical cutpoints is of little value. Importantly, there were no changes to patients’ dialysis prescriptions, provision of oral alkali, or changes in phosphate binder in response to these monthly laboratory values. Therefore, even if a clinician takes no action, approximately 50 % of bicarbonate values outside a normal range of 22–26 mEq/L will return to normal in the subsequent month. Using the mean value over 3 months modestly improves the predictive ability of low values, but not high ones. Regardless, even under the best circumstances, at least one-third of monthly or quarterly mean bicarbonate levels will change category with the next measurement. By comparison, consecutive monthly measurements of serum albumin, which is highly predictive of outcomes in ESRD patients [12] and is used to diagnose protein-energy wasting [13], were much more highly correlated.

This has clear implications for any intervention aimed at changing a hemodialysis patient’s metabolic acid-base status. First, the decision to intervene to raise or lower the serum bicarbonate should not be based on a single bicarbonate value, and even a 3-month mean may be insufficient. Second, if relying on monthly laboratory reports to determine the outcome, at least three measurements are required to reliably determine an effect. Thus, individualization of the dialysis prescription is a complex endeavor, and was not associated with improved mortality in a recent analysis of an international cohort [14]. Knowledge of a patient’s prior variability may inform the decision to intervene.

In our cohort, serum bicarbonate was inversely associated with several nutritional parameters. This is consistent with previous reports in HD patients which indicate that serum bicarbonate is partly determined by dietary intake and is a marker for nutritional status in ESRD patients [1, 2, 15–17]. Thus, one would expect that the variability in monthly serum bicarbonate measures would be related to patient nutrition. Our data point to non-linear associations of several nutritional parameters with bicarbonate variability, suggesting that patients at both ends of the nutritional spectrum exhibit the greatest variability. Although we also hypothesized that dialysis adequacy and interdialytic weight gain would be important determinants of bicarbonate variability, our findings did not support this. The lack of an association with Kt/V may be due to confounding by body size, which would also determine the volume of distribution of bicarbonate. Similarly, residual confounding related to nutritional factors and medication compliance may explain the lack of an association with interdialytic weight gain. Alternatively, the use of ultrafiltration volume as a surrogate for IDWG may have introduced imprecision into the measure and limited our ability to detect an association.

A number of previous studies have reported lower serum bicarbonate values with sevelamer hydrochloride use [18–21]. In our cohort, patients taking >6 pills per day of sevelamer hydrochloride had lower bicarbonate levels over time compared with no binder use, and there was a trend toward higher bicarbonate levels in patients taking binders containing base precursors. Binder use, regardless of type, was associated with lower variability over time. We hypothesize that binder use reduced bicarbonate variability by increasing the fraction of dietary acid that did not change from month to month.

We also noted an association of access type with monthly bicarbonate variability. The results of the time-stratified models suggest that use of an access other than an AVF is associated with greater variability, and that after 12 months, catheter use may be associated with the greatest variability. Access-related issues that affect dialysis adequacy and could also lead to missed treatments and hospitalizations are the most likely explanation for these findings. Access type, especially catheter use, could also be a marker for patients with poorer or more variable nutritional status.

We cannot account for the amount of variability due to sample handling and delays in measurement [22, 23]. However, this is unlikely to account for all the variability that we observed. Values measured at a central laboratory may be falsely low for a variety of reasons [24], although it has been suggested that this is a rare event [25]. Also, if falsely low values occurred frequently, we might expect to see the greatest variability among low serum bicarbonate measurements, but in our cohort high values were associated with the highest variability in multivariable analyses and were the least likely to be confirmed on a subsequent measurement. Furthermore, the independent associations of several clinical and laboratory values with bicarbonate variability suggest that patient-level factors contribute as well. Simultaneous measurement in a local laboratory would nevertheless have provided a useful comparison. Regardless, our findings remain clinically relevant to the day-to-day practice of managing patients’ dialysis prescriptions as providers rely on the monthly laboratory reports as the only data to guide their clinical decision-making.

Several other limitations should be noted. Data were not available for residual renal function, which could explain some of the decline in variability over time. Phosphate binder prescriptions were included in our models but without information on medication adherence. We could not account for any effect of dialysis shift in our analyses, nor did we have information on treatment adherence. Few data were available regarding respiratory function, but as there would be little or no metabolic compensation, the effect on serum bicarbonate would be minor. Lastly, the dietary patterns of our patients may not generally reflect other geographical regions and could limit the generalizability of our findings.

A number of additional questions remain unanswered. The magnitude of variability should be verified in larger cohorts, along with further examination of the effect of early and late dialysis vintage on variability. Future studies should also use local lab measurements to determine how much variability is accounted for by measurement error. It will be important to determine whether bicarbonate variability changes around the time of a hospitalization, and whether variability is predictive of outcomes. Finally, a more detailed understanding of the role of organic anion generation and other physiologic factors in modulating bicarbonate variability is needed [8].

## Conclusions

The pre-dialysis serum bicarbonate values on monthly laboratory reports in hemodialysis patients are highly variable. Further study is needed before they can be relied on as the basis for clinical intervention, especially as this relates to correcting high values.

## Additional file

Additional file 1: Table S1. Baseline characteristics by quartiles of baseline serum bicarbonate. **Table S2.** Transition matrix showing the number of serum bicarbonate measurements in a clinical category in a given month and in the subsequent month. **Figure S1.** Predicting next month’s serum bicarbonate level, within tertiles of 90-day mean serum bicarbonate. For a single monthly serum bicarbonate measurement falling within the low (<22 mEq/L), normal (22–26 mEq/L), and high (>26 mEq/L) categories defined by clinical cutpoints, the percentage of measurements in the following month that remain in the same category or change category are shown within tertiles of the mean serum bicarbonate during the first 90 days after admission to the dialysis unit: ≤22 mEq/L (*n* = 62), 22.25 – 24 mEq/L (*n* = 65), ≥24.25 mEq/L (*n* = 54). Percentages may not sum to exactly 100 % due to rounding. **Figure S2.** Predicting next month’s serum bicarbonate level, within tertiles of variability value. For a single monthly serum bicarbonate measurement falling within the low (<22 mEq/L), normal (22–26 mEq/L), and high (>26 mEq/L) categories defined by clinical cutpoints, the percentage of measurements in the following month that remain in the same category or change category are shown within tertiles of the mean value of VV (Variability Value) for each patient during follow-up: Low (mean VV ≤2.18 mEq/L), Medium (mean VV = 2.19 to 2.75 mEq/L), and High (mean VV ≥2.76 mEq/L). Percentages may not sum to exactly 100 % due to rounding. **Table S3.** Multivariable-adjusted associations of clinical and laboratory characteristics with serum bicarbonate variability over time within and after the first 6 months. (DOCX 67 kb)

## Abbreviations

AVF: Arteriovenous fistula
AVG: Arteriovenous graft
ESRD: End-stage renal disease
HD: Hemodialysis
ICC: Intraclass correlation coefficient
IDWG: Interdialytic weight gain
nPCR: Normalized protein catabolic rate
spKt/V: Single pool Kt/V
VV: Variability value
WBC: White blood cell
