# Supplementary material for: Variability in monthly serum bicarbonate measures in hemodialysis patients: a cohort study
Source: BMC Nephrol. 2015 Dec 21;16:214. doi: 10.1186/s12882-015-0206-2 (PMC4687073; doi:10.1186/s12882-015-0206-2)
Supplement: Additional file 1: Table S1. — Baseline characteristics by quartiles of baseline serum bicarbonate. Table S2. Transition matrix showing the number of serum bicarbonate measurements in a clinical category in a given month and in the subsequent month. Figure S1. Predicting next month’s serum bicarbonate level, within tertiles of 90-day mean serum bicarbonate. For a single monthly serum bicarbonate measurement falling within the low (<22 mEq/L), normal (22–26 mEq/L), and high (>26 mEq/L) categories defined by clinical cutpoints, the percentage of measurements in the following month that remain in the same category or change category are shown within tertiles of the mean serum bicarbonate during the first 90 days after admission to the dialysis unit: ≤22 mEq/L (n = 62), 22.25 – 24 mEq/L (n = 65), ≥24.25 mEq/L (n = 54). Percentages may not sum to exactly 100 % due to rounding. Figure S2. Predicting next month’s serum bicarbonate level, within tertiles of variability value. For a single monthly serum bicarbonate measurement falling within the low (<22 mEq/L), normal (22–26 mEq/L), and high (>26 mEq/L) categories defined by clinical cutpoints, the percentage of measurements in the following month that remain in the same category or change category are shown within tertiles of the mean value of VV (Variability Value) for each patient during follow-up: Low (mean VV ≤2.18 mEq/L), Medium (mean VV = 2.19 to 2.75 mEq/L), and High (mean VV ≥2.76 mEq/L). Percentages may not sum to exactly 100 % due to rounding. Table S3. Multivariable-adjusted associations of clinical and laboratory characteristics with serum bicarbonate variability over time within and after the first 6 months. (DOCX 67 kb) [file 12882_2015_206_MOESM1_ESM.docx]

**Supplementary Material**

**Table S1. Baseline characteristics by quartiles of baseline serum bicarbonate**

**Table S2. Transition matrix showing the number of serum bicarbonate measurements in a clinical category in a given month and in the subsequent month**

**Table S3. Multivariable-adjusted associations of clinical and laboratory characteristics with serum bicarbonate variability over time within and after the first 6 months**

**Figure S1. Predicting next month’s serum bicarbonate level, within tertiles of 90-day mean serum bicarbonate.** For a single monthly serum bicarbonate measurement falling within the low (<22 mEq/L), normal (22-26 mEq/L), and high (>26 mEq/L) categories defined by clinical cutpoints, the percentage of measurements in the following month that remain in the same category or change category are shown within tertiles of the mean serum bicarbonate during the first 90 days after admission to the dialysis unit: ≤22 mEq/L (n=62), 22.25 – 24 mEq/L (n=65), ≥24.25 mEq/L (n=54). Percentages may not sum to exactly 100% due to rounding.

**Figure S2. Predicting next month’s serum bicarbonate level, within tertiles of variability value.** For a single monthly serum bicarbonate measurement falling within the low (<22 mEq/L), normal (22-26 mEq/L), and high (>26 mEq/L) categories defined by clinical cutpoints, the percentage of measurements in the following month that remain in the same category or change category are shown within tertiles of the mean value of VV (Variability Value) for each patient during follow-up: Low (mean VV ≤2.18 mEq/L), Medium (mean VV=2.19 to 2.75 mEq/L), and High (mean VV ≥2.76 mEq/L). Percentages may not sum to exactly 100% due to rounding.

| **Table S1. Baseline characteristics by quartiles of baseline serum bicarbonate^a^** | | | | | |
| --- | --- | --- | --- | --- | --- |
| Serum Bicarbonate (mEq/L) | 16.7 - 21.7 | 21.8 – 23.0 | 23.1 - 24.5 | 24.6 – 32.8 | p |
| Number | 46 | 51 | 41 | 43 |  |
| Age (years) | 50.9 ± 16.4 | 58.1 ± 15.8 | 55.8 ± 18.3 | 59.5 ± 15.3 | 0.07 |
| Female – n (%) | 13 (28.3) | 21 (41.2) | 20 (48.8) | 20 (46.5) | 0.20 |
| Race/ethnicity – n (%) |  |  |  |  | 0.09 |
| *Black* | 16 (34.8) | 23 (45.1) | 17 (41.5) | 23 (53.5) |  |
| *White* | 9 (19.6) | 1 (2.0) | 6 (14.6) | 6 (14.0) |  |
| *Hispanic/Other* | 21 (45.7) | 27 (52.9) | 18 (43.9) | 14 (32.6) |  |
| Hypertension– n (%) | 44 (95.7) | 50 (98.0) | 40 (97.6) | 42 (97.7) | 0.9 |
| Diabetes Mellitus – n (%) | 25 (54.4) | 31 (60.8) | 25 (61.0) | 27 (62.8) | 0.86 |
| Cardiovascular Disease – n (%) | 25 (54.4) | 32 (62.8) | 28 (68.3) | 31 (72.1) | 0.33 |
| Smoking – n (%) |  |  |  |  | 0.31 |
| *Current* | 11 (23.9) | 4 (7.8) | 4 (9.8) | 5 (11.6) |  |
| *Former* | 6 (13.0) | 12 (23.5) | 8 (19.5) | 7 (16.3) |  |
| *Never* | 10 (21.7) | 18 (35.3) | 10 (24.4) | 15 (34.9) |  |
| *Unknown* | 19 (41.3) | 17 (33.3) | 19 (46.3) | 16 (37.2) |  |
| Body Mass Index (kg/m^2^) – n (%) |  |  |  |  | 0.9 |
| *Normal/Low* (16.3 - 24.9) | 19 (41.3) | 17 (33.3) | 15 (36.6) | 15 (34.9) |  |
| *Overweight* (25 - 29.9) | 17 (37.0) | 19 (37.3) | 13 (31.7) | 15 (34.9) |  |
| *Obese* (≥ 30) | 10 (21.7) | 15 (29.4) | 13 (31.7) | 13 (30.2) |  |
| ESRD Etiology – n (%) |  |  |  |  | 0.50 |
| *Diabetes* | 21 (45.7) | 26 (51.0) | 17 (41.5) | 23 (53.5) |  |
| *Hypertension* | 11 (23.9) | 16 (31.4) | 11 (26.8) | 7 (16.3) |  |
| *Other/Unknown* | 14 (30.4) | 9 (17.7) | 13 (31.7) | 13 (30.2) |  |
| Initial Access Type – n (%) |  |  |  |  | 0.08 |
| *Catheter* | 13 (28.3) | 22 (43.1) | 16 (39.0) | 24 (55.8) |  |
| *AVF* | 24 (52.2) | 14 (27.5) | 16 (39.0) | 12 (27.9) |  |
| *AVG* | 9 (19.6) | 15 (29.4) | 9 (22.0) | 7 (16.3) |  |
| Medication use – n (%) |  |  |  |  |  |
| *Diuretic* | 8 (17.4) | 8 (15.7) | 6 (14.6) | 3 (7.0) | 0.50 |
| *ACE inhibitor or ARB* | 21 (45.7) | 10 (19.6) | 15 (36.6) | 17 (39.5) | 0.05 |
| *Beta blocker* | 30 (65.2) | 41 (80.4) | 32 (78.1) | 30 (69.8) | 0.31 |
| *Calcium Channel Blocker* | 31 (67.4) | 31 (60.8) | 26 (63.4) | 21 (48.8) | 0.32 |
| *Statin* | 20 (43.5) | 27 (52.9) | 20 (48.8) | 26 (60.5) | 0.44 |
| *Proton Pump Inhibitor* | 14 (30.4) | 14 (27.5) | 11 (26.8) | 12 (27.9) | 0.9 |
| *H2 Blocker* | 1 (2.2) | 5 (9.8) | 3 (7.3) | 1 (2.3) | 0.28 |
| Phosphate binder– n (%) |  |  |  |  | 0.39 |
| *Sevelamer HCl* | 21 (45.7) | 27 (52.9) | 12 (29.3) | 16 (37.2) |  |
| *Other Binder* | 17 (37.0) | 17 (33.3) | 20 (48.8) | 17 (39.5) |  |
| *None* | 8 (17.4) | 7 (13.7) | 9 (22.0) | 10 (23.3) |  |
| Epoetin alpha dose (Units) |  |  |  |  | 0.55 |
| *≤5000* | 10 (21.7) | 16 (31.4) | 14 (34.2) | 17 (39.5) |  |
| *>5000 - ≤10,000* | 28 (60.9) | 29 (56.9) | 21 (51.2) | 18 (41.9) |  |
| *≥10,000* | 8 (17.4) | 6 (11.8) | 6 (14.6) | 8 (18.6) |  |
| Interdialytic weight gain (kg) | 3.20 ± 0.91 | 2.79 ± 0.93 | 2.48 ± 0.94 | 2.56 ± 0.78 | <0.001 |
| Dialysis treatment time (min) | 227.8 ± 19.4 | 231.1 ± 18.9 | 226.0 ± 16.7 | 228.2 ± 16.8 | 0.60 |
| Blood flow rate (mL/min) | 382 ±35 | 383 ± 27 | 385 ± 31 | 375 ± 30 | 0.37 |
| Dialysate flow rate (mL/min) | 602 ± 45 | 604 ± 25 | 610 ± 34 | 598 ± 27 | 0.40 |
| Serum potassium (mEq/L) | 4.8 ± 0.6 | 4.7 ± 0.7 | 4.6 ± 0.6 | 4.4 ± 0.6 | 0.008 |
| Serum albumin (g/dL) | 3.8 ± 0.4 | 3.7 ± 0.4 | 3.8 ± 0.4 | 3.5 ± 0.5 | 0.005 |
| Serum calcium (mg/dL) | 8.8 ± 0.5 | 8.9 ± 0.6 | 8.8 ± 0.8 | 8.9 ± 0.6 | 0.92 |
| Serum phosphate (mg/dL) | 5.9 ± 1.3 | 5.0 ± 1.0 | 5.0 ± 0.8 | 4.4 ± 1.0 | <0.001 |
| Serum hemoglobin (g/dL) | 10.8 ± 1.2 | 10.9 ± 0.9 | 10.9 ± 1.1 | 10.5 ± 1.4 | 0.40 |
| Serum creatinine (mg/dL) | 8.5 ± 2.7 | 8.3 ± 2.7 | 7.8 ± 2.8 | 7.4 ± 3.3 | 0.30 |
| WBC (10^3^/mm^3^) | 7.6 ± 1.7 | 7.7 ± 2.3 | 7.5 ± 2.2 | 7.0 ± 2.1 | 0.33 |
| nPCR (g/kg/day) | 0.87 ± 0.18 | 0.84 ± 0.16 | 0.84 ± 0.17 | 0.72 ± 0.18 | <0.001 |
| spKt/V | 1.5 ± 0.3 | 1.6 ± 0.3 | 1.6 ± 0.3 | 1.6 ± 0.3 | 0.18 |

Data for continuous variables are expressed as mean ± SD.

^a^ Baseline serum bicarbonate is the mean value of all measurements within the first 90 days after admission to the dialysis unit.

| **Table S2. Transition matrix showing the number of serum bicarbonate measurements in a clinical category in a given month and in the subsequent month** | | | | |
| --- | --- | --- | --- | --- |
|  | **Clinical Category the Next Month** | | |  |
| **Clinical Category** | Low (<22 mEq/L) | Normal (22-26 mEq/L) | High (>26 mEq/L) | Total |
| Low (<22 mEq/L) | 754 | 536 | 20 | 1310 |
| Normal (22-26 mEq/L) | 519 | 1525 | 210 | 2254 |
| High (>26 mEq/L) | 20 | 208 | 118 | 346 |
| Total | 1293 | 2269 | 348 | 3910 |

| **Table S3. Multivariable-Adjusted Associations of clinical and laboratory characteristics with serum bicarbonate variability over time within and after the first 6 months** | | | | |
| --- | --- | --- | --- | --- |
|  | ≤ 6 months | | > 6 months | |
|  | Coefficient (95% CI) | p | Coefficient (95% CI) | p |
| Age (per 10 years) | -0.01 (-0.04 to 0.02) | 0.39 | **-0.03 (-0.05 to 0.00)** | 0.02 |
| Male | **-0.11 (-0.20 to -0.02)** | 0.01 | 0.01 (-0.05 to 0.08) | 0.72 |
| Race/ethnicity |  |  |  |  |
| *White* | -0.05 (-0.18 to 0.08) | 0.46 | -0.03 (-0.13 to 0.07) | 0.51 |
| *Hispanic/Other* | -0.02 (-0.11 to 0.07) | 0.65 | -0.03 (-0.10 to 0.03) | 0.31 |
| BMI |  |  |  |  |
| *Overweight* | 0.04 (-0.05 to 0.14) | 0.35 | -0.01 (-0.08 to 0.06) | 0.77 |
| *Obese* | 0.02 (-0.09 to 0.12) | 0.75 | -0.03 (-0.10 to 0.05) | 0.49 |
| Dialysis access |  |  |  |  |
| *AVG* | 0.02 (-0.07 to 0.11) | 0.67 | **0.08 (0.02 to 0.14)** | 0.01 |
| *Tunneled catheter* | -0.02 (-0.10 to 0.06) | 0.68 | **0.10 (0.03 to 0.18)** | 0.01 |
| Etiology of ESRD |  |  |  |  |
| *Diabetes* | -0.04 (-0.14 to 0.06) | 0.45 | -0.02 (-0.10 to 0.05) | 0.56 |
| *Other/Unknown* | -0.04 (-0.16 to 0.08) | 0.50 | -0.06 (-0.14 to 0.03) | 0.19 |
| Cardiovascular Disease | 0.05 (-0.04 to 0.14) | 0.29 | 0.04 (-0.03 to 0.11) | 0.24 |
| Phosphate binder |  |  |  |  |
| *>6 pills/day acid precursor* | 0.03 (-0.13 to 0.18) | 0.73 | -0.10 (-0.21 to 0.00) | 0.06 |
| *>3-6 pills/day acid precursor* | 0.02 (-0.11 to 0.14) | 0.79 | **-0.12 (-0.23 to -0.02)** | 0.02 |
| *≤3 pills/day acid precursor* | -0.06 (-0.16 to 0.04) | 0.27 | **-0.14 (-0.24 to -0.04)** | 0.01 |
| *≤3 pills/day base precursor* | -0.01 (-0.10 to 0.09) | 0.88 | -0.09 (-0.18 to 0.01) | 0.07 |
| *>3-6 pills/day base precursor* | -0.02 (-0.13 to 0.10) | 0.74 | **-0.13 (-0.23 to -0.03)** | 0.01 |
| *>6 pills/day base precursor* | 0.03 (-0.11 to 0.18) | 0.67 | **-0.12 (-0.22 to -0.03)** | 0.01 |
| Epoetin alpha dose (Units) |  |  |  |  |
| *≤5000* | -0.03 (-0.11 to 0.05) | 0.43 | **0.09 (0.05 to 0.13)** | <0.001 |
| *>5000 - ≤10,000* | -0.01 (-0.09 to 0.06) | 0.76 | 0.04 (-0.02 to 0.09) | 0.19 |
| *≥10,000* | -0.06 (-0.16 to 0.04) | 0.21 | 0.15 (0.07 to 0.22) | <0.001 |
| Serum bicarbonate (mEq/L) |  |  |  |  |
| *<22* | **0.21 (0.15 to 0.28)** | <0.001 | **0.08 (0.04 to 0.12)** | <0.001 |
| *>26* | **0.29 (0.19 to 0.38)** | <0.001 | **0.22 (0.16 to 0.28)** | <0.001 |
| Serum albumin (g/dL) | **0.13 (0.04 to 0.22)** | 0.01 | -0.03 (-0.10 to 0.04) | 0.46 |
| nPCR (g/kg/day) | -0.37 (-1.10 to 0.36) | 0.32 | **-0.46 (-0.82 to -0.11)** | 0.01 |
| nPCR^2^ ((g/kg/day)^2^) | 0.19 (-0.19 to 0.57) | 0.32 | **0.21 (0.05 to 0.37)** | 0.01 |
| spKt/V | -0.03 (-0.13 to 0.07) | 0.53 | 0.06 (-0.01 to 0.13) | 0.10 |
| K^+^ (mEq/L) | -0.10 (-0.43 to 0.23) | 0.54 | **-0.26 (-0.48 to -0.04)** | 0.02 |
| K^+2^ ((mEq/L)^2^) | 0.01 (-0.02 to 0.04) | 0.50 | **0.03 (0.01 to 0.05)** | 0.01 |
| Hemoglobin | **-0.04 (-0.06 to -0.02)** | <0.001 | 0.00 (-0.02 to 0.01) | 0.61 |
| Calcium (mg/dL) | **-0.44 (-0.82 to -0.06)** | 0.02 | -0.20 (-0.47 to 0.08) | 0.17 |
| Calcium^2^ ((mg/dL)^2^) | **0.02 (0.00 to 0.05)** | 0.03 | 0.01 (0.00 to 0.03) | 0.12 |
| Phosphorus (mg/dL) | -0.01 (-0.03 to 0.02) | 0.60 | **0.02 (0.01 to 0.03)** | <0.001 |
| Serum creatinine (mg/dL) | 0.00 (-0.01 to 0.02) | 0.64 | **0.01 (0.00 to 0.02)** | 0.02 |
| WBC | 0.01 (0.00 to 0.03) | 0.13 | 0.00 (-0.01 to 0.01) | 0.73 |
| IDW gain (kg) |  |  |  |  |
| *>1-3* | -0.04 (-0.13 to 0.06) | 0.47 | 0.04 (-0.03 to 0.12) | 0.24 |
| *>3-7* | -0.04 (-0.15 to 0.06) | 0.43 | 0.07 (-0.01 to 0.15) | 0.07 |
| Time (months) |  |  |  |  |
| *>3-6* | **-0.06 (-0.11 to 0.00)** | 0.04 | …. |  |
| *>6-12* | **….** |  | ref |  |
| *>12-24* | **….** |  | -0.03 (-0.07 to 0.01) | 0.18 |
| *>24* | **….** |  | -0.03 (-0.08 to 0.02) | 0.18 |

Abbreviations: CI, confidence interval; BMI, body-mass index; AVG, arteriovenous graft; ESRD, end-stage renal disease; nPCR, normalized protein catabolic rate; spKt/V, single-pool Kt/V; WBC, white blood cells; IDW, interdialytic weight gain.

Models included all variables listed in the Table. Reference categories are female for sex; Black for race/ethnicity; normal/underweight for BMI; arteriovenous fistula (AVF) for dialysis access; hypertension for etiology of ESRD; no binder use for phosphate binder; no epoetin alpha use for epoetin alpha dose; 22-26 mEq/L for serum bicarbonate; 0-1kg for interdialytic weight gain; ≤3 months for time in the ≤6 months models, and >6-12 months in the >6 months model.

|  | **Figure S1. Predicting Next Month’s Serum Bicarbonate Level, Within Tertiles of 90-Day Mean Serum Bicarbonate^*^** | | |
| --- | --- | --- | --- |
|  | **If this month’s serum bicarbonate is…** | | |
| (mEq/L) | **Low**  (<22) | **Normal**  (22-26) | **High**  (>26) |
|  | **Next month it will…** | | |
| Mean 90-day Serum Bicarbonate ≤22 |  |  |  |
| Mean 90-day Serum Bicarbonate 22.25-24 |  |  |  |
| Mean 90-day Serum Bicarbonate ≥24.25 |  |  |  |

|  | **Figure S2. Predicting Next Month’s Serum Bicarbonate Level, Within Tertiles of Variability Value^*^** | | |
| --- | --- | --- | --- |
|  | **If this month’s serum bicarbonate is…** | | |
| (mEq/L) | **Low**  (<22) | **Normal**  (22-26) | **High**  (>26) |
|  | **Next month it will…** | | |
| Low Variability |  |  |  |
| Medium Variability |  |  |  |
| High Variability |  |  |  |
